# Editorial: Brain-inspired computing: Neuroscience drives the development of new electronics and artificial intelligence

**DOI:** 10.3389/fncel.2022.1115395

**Published:** 2022-12-20

**Authors:** Daniela Gandolfi, Francesco Maria Puglisi, Alexander Serb, Michele Giugliano, Jonathan Mapelli

**Affiliations:** ^1^Department of Biomedical, Metabolic and Neural Sciences, University of Modena and Reggio Emilia, Modena, Italy; ^2^Department of Engineering “Enzo Ferrari,” University of Modena and Reggio Emilia, Modena, Italy; ^3^Center for Neuroscience and Neurotechnology, University of Modena and Reggio Emilia, Modena, Italy; ^4^Centre for Electronics Frontiers, School of Engineering, University of Edinburgh, Edinburgh, United Kingdom; ^5^Neuroscience Area, International School of Advanced Studies (SISSA), Trieste, Italy

**Keywords:** neuromorphic, neuro-inspired computing, computational neuroscience, bio-inspired artificial intelligence, electronic

The advent of artificial intelligence (AI) applications in everyday life has continuously raised the need for the deployment of advanced machine learning systems like artificial neural networks, which in increasingly numerous tasks outperform humans. Moreover, the development of humanoid robotics platforms showing intelligent behaviors has boosted research on engineering systems that mimic neural functions. Nonetheless, solutions based on conventional hardware with limited energy efficiency are poorly sustainable, require repeated training cycles, depend on supervised learning rules, and most importantly must be trained offline on vert large datasets to perform online on the desired tasks. All these factors, severely limit the pervasive distribution of AI sustainable solutions. In the recent years, a large number of industrial applications based on classic computational paradigms and conventional technologies have emerged. In this scenario, a significant new opportunity is represented by alternative, sustainable strategies inspired by the ways the human brain works, known as *neuromorphic*.

The term neuromorphic is strongly interdisciplinary and encompasses many fields spanning from material sciences to electronic architectures and software models, all sharing a common framework dictated by some analogy with brain functions. On the hardware side, the development of bio-inspired microelectronics dates back to the 80's (Mead, 1989) and was grounded in the analogy between the biophysics at work in biological neuronal membranes and the charge carriers behavior of semiconductor transistors in sub-threshold regime (Furber, 2016). Many scientific works have appeared since those initial seminal papers, testifying progresses along four main directions:

i) *Materials:* Novel nanomaterials proposed as candidates for neuromorphic computing devices, showing functionalities that better mimic neuronal behaviors (Sangwan and Hersam, 2020);
ii) *Synapses:* Memristive devices, as alternatives to conventional data storage concepts, capable of reproducing peculiar characteristics of biological synaptic long-term plasticity in both supervised (Florini et al., 2022) and unsupervised regimes (Serb et al., 2016);
iii) *Neurons:* Silicon neurons as the main building block of novel architectures, implementing systems reacting in real-time or bidirectional brain-machine interfaces, where the complexity of their design scales up with the level of detail of neuronal representation (Indiveri et al., 2011);
iv) *Architecture:* Spiking neural networks (SNN) as sustainable solutions, mimicking brain circuits operations, to efficiently perform distributed computation. The development of silicon architectures supporting SNN is one of the main cores of neuromorphic computing research (George et al., 2020). In a broader perspective, electronic architecture in the context of AI is a system that has been designed and optimized for implementing in hardware conventional artificial neural networks or alternatively to emulate spiking networks (Rose et al., 2021).

On the software side, much of the effort in the development of neuromorphic solutions has been devoted to the implementation of AI algorithms that could draw inspiration from neuronal circuit architectures. Conversely, it is widely accepted that despite an impressive number of papers published in the last two decades proposing AI solutions based on ANN, the growth rate of deployed applications is decreasing. There is a general agreement that alternatives must be explored and the brain, through its spike-based operations, still remains the main target given its impressive computational capacity on top of limited metabolic energy budgets.

In most cases, these research directions are undertaken separately despite a common thread, inspired by the same neural principles. This joint Frontiers in Cellular Neuroscience and Frontiers in Neuroscience topic was launched to gather new developments in the neuromorphic field and, at the same time, to explore how extensive reciprocal contaminations are. The first example of this attempt is shown in the work by Lee et al., which introduces an analogy between one of the most widely employed techniques adopted to regularize artificial neural networks (ANN) and a real biological architecture. Authors propose that the serotoninergic axons satisfy most of the requirements for the ANN parallelization and as a proof-of-concept they demonstrate that serotoninergic fibers through their stochasticity can potentially implement a dropout mechanism that supports neuroplasticity. This example suggests how coupling a deeper investigation of neuronal, functional and structural properties can lead to improved AI performance. In an attempt of practical applications for bio-inspired SNN, Vijayan and Diwakar present a cerebellum-inspired SNN architecture that can efficiently perform pattern classification with an accuracy comparable to state-of-the-art machine learning algorithms. The same algorithm, inspired by the physiology of cerebellar network, allowed them to reconstruct the kinematic parameters of a robotic arm to be controlled with a low error rate. This work shows the potential of SNNs and their versatility for different applications. On the same theme, Bittar and Garner fully explore the potential of different spiking neuron models in SNN, showing how the integration of different network architectures and training algorithms lead to intriguing perspectives in classification tasks such as speech recognition. Moving to the hardware level, Baroni et al. offer an energy efficient electronic architecture, based on resistive switching memory crossbars that forms a convenient primitive for matrix-vector multiplication in a single computational step. A similar improvement in performances can boost ANNs' speed of operation, with ideal applications in big-data related fields like biomedical or genomics research.

We thank the authors for their excellent contributions to this Research Topic and we hope that this collection provides the first step for a series of cross-disciplinary works on brain-inspired computing, combining software and hardware, and more generally linking conventional AI to bio-inspired systems. We look forward to continuing the discussion in the field.

## Author contributions

JM wrote the first draft of the manuscript. All authors contributed equally to the editing of the Research Topic and conceptualization of the manuscript. All authors contributed to revisions and approved the submitted version.
